# Reliability of Temporal Summation of Pain in Healthy and Clinical Populations: A Systematic Review and Meta‐Analysis

**DOI:** 10.1002/ejp.70097

**Published:** 2025-08-08

**Authors:** Kexun Kenneth Chen, Paul Rolan, Mark Rowland Hutchinson, Rutger Marinus Johannes de Zoete

**Affiliations:** ^1^ School of Allied Health Science and Practice, Faculty of Health and Medical Science The University of Adelaide Adelaide South Australia Australia; ^2^ School of Biomedicine, Faculty of Health and Medical Sciences The University of Adelaide Adelaide South Australia Australia; ^3^ Institute for Photonics and Advanced Sensing, The University of Adelaide Adelaide South Australia Australia

## Abstract

**Background:**

Temporal summation of pain (TSP) is a dynamic quantitative sensory test reflecting pain facilitation. Variability in TSP paradigms challenges cross‐study comparisons and raises concerns about reliability. This systematic review and meta‐analysis evaluated the reliability of TSP in healthy and clinical populations.

**Methods:**

Four databases were searched for peer‐reviewed studies up to July 2024. Risk of bias and study quality were assessed. A random‐effects meta‐analysis was conducted to estimate intraclass correlation coefficients (ICC).

**Results:**

Twenty‐two studies met inclusion criteria, with data from healthy (*n* = 12) and clinical (*n* = 7) populations. TSP was assessed using absolute change (difference between first and last stimuli) and relative change (ratio of first to last). ICCs ranged from −0.31 to 0.97. In healthy participants, between‐session reliability was poor (ICC = 0.49, 95% CI: 0.40–0.58, *I* ^2^ = 81.4%, 35 estimates, 1208 subjects), while within‐session reliability was moderate (ICC = 0.69, 95% CI: 0.61–0.77, *I* ^2^ = 91.7%, 28 estimates, 728 subjects). Highest reliability was seen for within‐session testing using absolute change (ICC = 0.81, 95% CI: 0.74–0.88, *I* ^2^ = 0%). In clinical populations, between‐session reliability was moderate (ICC = 0.57, 95% CI: 0.44–0.69, *I* ^2^ = 80.8%, 13 estimates, 470 subjects); within‐session reliability was also moderate (ICC = 0.61, 95% CI: 0.46–0.76, *I* ^2^ = 89.6%, 10 estimates, 381 subjects). Study quality ranged from very good to excellent, with bias rated from doubtful to adequate.

**Conclusion:**

TSP demonstrates moderate reliability, influenced by stimulus parameters and population. Small‐area mechanical stimuli (e.g., pinprick), tested at 1 Hz using absolute change scores, yield the most reliable results. Further research is needed in clinical populations to better understand TSP mechanisms.

**Significance Statement:**

This review suggests that TSP is a reliable measure, with small contact area mechanical stimulus with absolute change calculation method, applied at a rate of 1 Hz, was found to be most reliable. Results highlights the need for standardisation, and consistency in data reporting. While most TSP paradigms were found to have moderate to good reliability, consideration should also be given when translating experimental TSP testing to clinical routine assessment.

**Trial Registration:**

PROSPERO number: CRD42024566623

## 1 Introduction

The central nervous system is responsible for the perception of pain when there is peripheral nociceptive stimulation, either reducing the pain intensity (pain inhibition) or increasing the pain intensity (pain facilitation) (Staud 2012). Conditioned pain modulation (CPM) is used to assess the endogenous pain inhibitory pathway in humans, commonly known as the ‘pain inhibit pain’ phenomenon (Kennedy et al. 2016; Nuwailati et al. 2022). It is believed to represent the human behavioural correlate of diffuse noxious inhibitory control in animals (Kennedy et al. 2016; Nuwailati et al. 2022). Temporal summation of pain (TSP) is commonly used to assess the efficacy of endogenous pain facilitation in humans, and is commonly referred to as the physiological correlate of wind‐up (Eide 2000).

TSP measures are thought to assess excitatory neural activity in the dorsal horn of the spinal cord and have been found to be mediated by central nervous system mechanisms (Arendt‐Nielsen et al. 2018). TSP assesses the human pain facilitatory mechanism, an increased pain experience in response to a rapid succession of identical noxious stimuli. TSP has been used to assess pain facilitation in various clinical studies, and an increased magnitude of TSP has been found in various chronic pain conditions, such as chronic low back pain (Arendt‐Nielsen et al. 2018), fibromyalgia (Arendt‐Nielsen et al. 2018; Staud et al. 2003) and knee osteoarthritis (Goodin et al. 2014).

Despite the wide use of TSP in research studies, TSP paradigms remain unstandardised, with diverse paradigms utilised to assess TSP (Kielstra et al. 2024). The methodological basis of TSP testing comprises a single brief noxious test stimulus, followed by a series of repetitive identical noxious stimuli at fixed intervals (Rolke et al. 2006). The participant subjectively rates the pain intensity of the single and repeated stimuli, and TSP is then calculated based on either the absolute (i.e., difference between first and last stimuli) or relative (i.e., ratio between first and last stimuli) change between the two pain ratings (Rolke et al. 2006). Though the methodological basis of assessing TSP is well agreed (Rolke et al. 2006), there is a large heterogeneity in the design of experimental TSP protocols, such as instruments used, type of stimuli, inter‐stimuli intervals, stimuli intensities used, calculations and test locations (Kielstra et al. 2024). With such diverse paradigms, comparison of TSP outcomes between studies is difficult and may lead to unreliable interpretation of results.

Recent work has begun in developing recommendations and a gold standard for TSP assessments (Kielstra et al. 2024). A recent scoping review identified and mapped key elements of the TSP assessment (Nuwailati et al. 2022). From the review, the main elements were identified: five different types of stimulus, 46 different instruments, 13 different test sites, 31 different inter‐stimulus intervals and 63 different calculation variations to quantify TSP (Nuwailati et al. 2022). While the review reported the diverse paradigms used to assess TSP, one key factor in forming recommendations for the assessment of TSP is its reliability. To our knowledge, two systematic reviews, including one meta‐analysis, were conducted to assess the reliability of CPM (Kennedy et al. 2016; Nuwailati et al. 2022), and recommendations for assessing and reporting CPM were published (Yarnitsky et al. 2015). However, such a reliability study for TSP is lacking. Therefore, this study aimed to assess the reliability of TSP, which may aid in providing more knowledge in paving the way for developing gold standards.

## 2 Methods

The protocol of this review, which was adapted from a previously published systematic review on the reliability of conditioned pain modulation (Kennedy et al. 2016; Nuwailati et al. 2022), was registered on PROSPERO. Findings are reported accordingly with the Preferred Reporting Items for Systematic Review and Meta‐Analyses (PRISMA) guidelines for systematic reviews (Page et al. 2021).

### 2.1 Literature Search

We searched PubMed, Embase and Web of Science databases from inception to 8 July 2024. The following search terms were used: temporal summation OR wind‐up OR dynamic sensory testing OR quantitative sensory testing (Data S1).

Inclusion criteria were as follows: (1) original studies in full‐text English; (2) adults (age: ≥ 18 years), healthy individuals or individuals with clinical conditions; (3) study design of repeated measures of pain modulation, specifically TSP or wind‐up, in two or more identical sessions, and studies that reported the TSP reliability; (4) measurement of TSP using one or more noxious stimuli to induce TSP effect, including measurement of pain rating of the single‐stimulus (or first) and repeated‐stimulus (or last stimulus); and (5) outcome measure includes estimates of TSP reliability (intraclass correlation coefficient, ICC). Two independent reviewers (K.C. & R.d.Z.) screened titles, abstracts and full text to determine study inclusion. Any disagreement on including a study in either of the screening stages was resolved through consensuses, or by consulting a third reviewer.

### 2.2 Data Extraction

Data was extracted by two independent reviewers using a pre‐piloted form. Any disagreements were resolved through consensus and, if needed, consultation with the third reviewer. Extracted information included study details (author, year), sample size, participant demographics (gender, mean age) and designation as healthy or clinical populations. In keeping consistent with the previous review on CPM reliability (Kielstra et al. 2024), we extracted TSP paradigm information including type of test stimulus (e.g., mechanical, thermal, electrical), tested body sites (e.g., hand, leg), fixed (i.e., all participants received the same test stimulus intensity, e.g., 256 mN pinprick) or individualised (i.e., each participant received a test stimulus intensity based on self‐reported pain rating, e.g., temperature at which participant report 7/10 NRS pain rating using a thermal stimulus) test stimulus intensity and inter‐stimulus interval (ISI). Reliability data extraction includes number of visits (i.e., testing conducted on different days), number of sessions (i.e., testing conducted per visit), interval between session visits, reliability measures of TSP effect and measure of response stability.

All reliability measures (e.g., intraclass correlation, ICC) estimates were stratified individually for each study (i.e., if a study reported ICC for each assessed body site, each body site's findings were extracted and analysed). Based on the ICC estimate, reliability is rated as poor (ICC < 0.5), moderate (ICC between 0.5 and 0.75), good (ICC between 0.75 and 0.9) and excellent (ICC > 0.90) (Koo and Li 2016).

### 2.3 Risk of Bias Assessment

The risk of bias and methodological quality of the included studies were assessed by two independent reviewers (K.C. & R.d.Z.). The Consensus‐based standards for the selection of health measurement instruments—risk of bias checklist (COSMIN‐ROB) tool was used to assess the methodological quality of studies on reliability and measurement error of outcome measurement instruments (Mokkink et al. 2024). COSMIN‐ROB for patient reported outcome measures (PROM) version 2.0 was used to assess the risk of bias. Studies were assessed on a 4‐point scale, ‘very good’, ‘adequate’, ‘doubtful’ and ‘inadequate’, with the overall rating of quality determined by the lowest rating. Next, the results of the reliability studies were rated against the criteria for good content validity, ‘sufficient (+), if ICC or (weighted) kappa or Pearson/Spearman correlation ≥ 0.70’, ‘insufficient (−), if ICC or (weighted) kappa or Pearson/Spearman correlation < 0.70’, or ‘indeterminate (?), if not enough information reported’.

Quality of individual studies was appraised using the quality appraisal for clinical measurement research report evaluation form (QACMRR) (Roy et al. 2011) and appraised independently by two reviewers (K.C. & R.d.Z). The evaluation criteria consist of 12 items: (1) thorough literature review to define the research question; (2) specific inclusion/exclusion criteria; (3) specific hypotheses; (4) appropriate scope of psychometric properties; (5) sample size; (6) follow‐up; (7) the authors referenced specific procedures for administration, scoring and interpretation of procedures; (8) measurement techniques were standardised; (9) data were presented for each hypothesis; (10) appropriate statistics‐point estimates; (11) appropriate statistical error estimates; and (12) valid conclusions and recommendations. A study's total score, reflecting its quality, was presented as a percentage score (i.e., sum of scores for each item divided by the number of items and multiplied by 100%). The quality summary of appraised study ranged from poor (0%–30%), fair (31%–50%), good (51%–70%), very good (71%–90%) and excellent (> 90%) (Roy et al. 2011).

### 2.4 Statistical Analysis

All descriptive data, including participants' age, number of visits, number of sessions, interval between sessions, type of stimulus used, ICC estimates and checklist findings were presented as mean ± standard deviation (SD), range of values, percent or score.

Meta‐analysis of reliability coefficients was conducted using STATA (StataCorp LLC. 2024. *StataNow/MP 18.5*. College Station, TX: StataCorp LLC.) with the meta package. Meta‐analyses were conducted using a random effects model, and coefficients were converted to Fisher's z‐transformed correlation values. Heterogeneity was deemed substantial if *I* ^2^ values were more than 50%, and subsequently, univariate meta‐regressions were performed to explain the source of heterogeneity. Negative ICC estimates were excluded from the analysis, as negative estimates are theoretically not possible, but negative estimates, if obtained, can be regarded as zero (Matheson 2019).

## 3 Results

**FIGURE 1:**
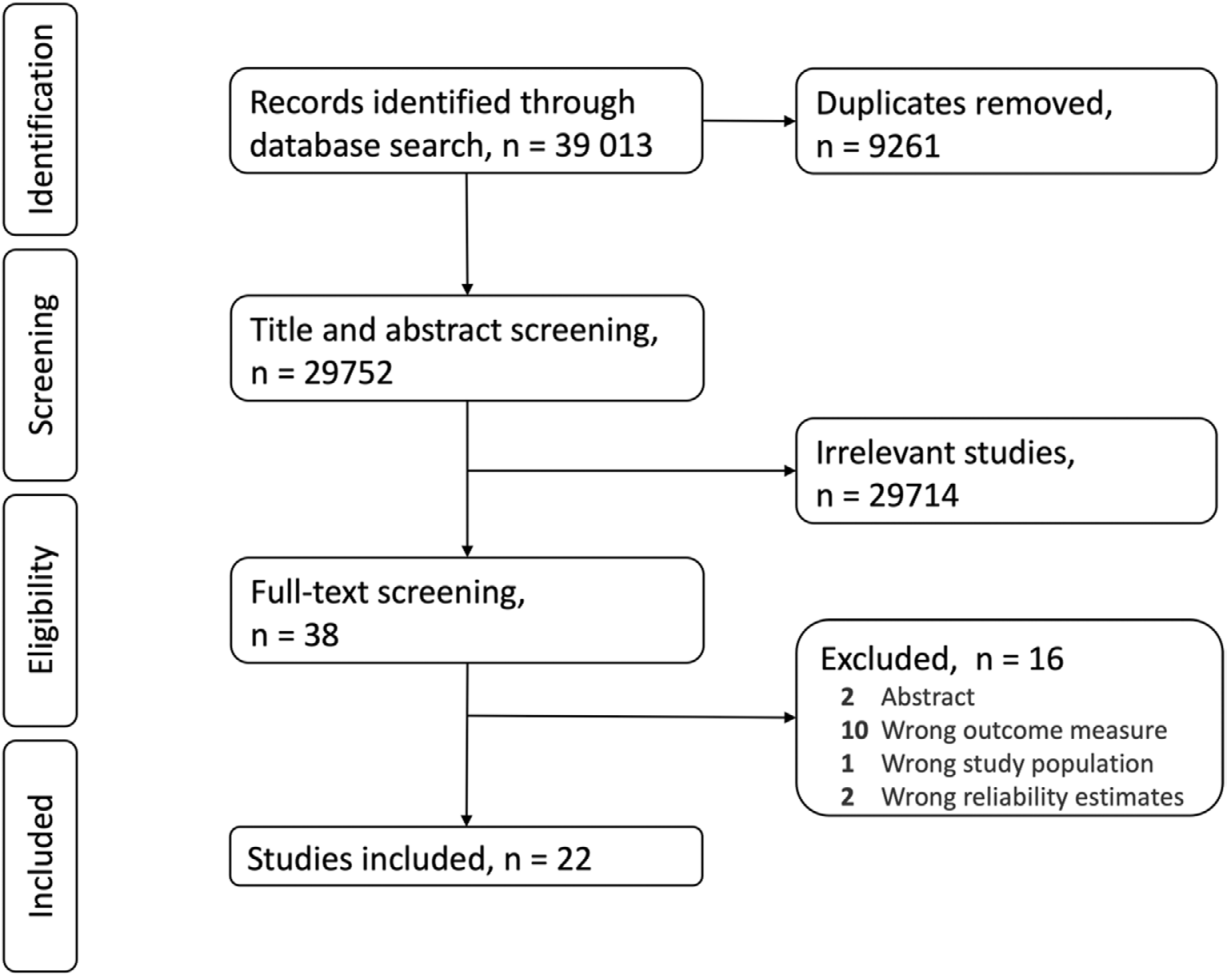
PRISMA study flow diagram.

The PRISMA flow diagram for the selection of included studies is illustrated in Figure 1. After title/abstract and full text assessed for eligibility, 22 (Baad‐Hansen et al. 2015; Biurrun Manresa et al. 2011; Brady et al. 2023; Cathcart et al. 2009; Costa et al. 2019, 2017; Dams et al. 2022; de la Coba et al. 2018; de Oliveira et al. 2023; De Vita et al. 2022; Graven‐Nielsen et al. 2015; Izumi et al. 2022; Knox et al. 2024; Kong et al. 2013; Mailloux et al. 2021; Marcuzzi et al. 2017; Middlebrook et al. 2020; Nothnagel et al. 2017; Othman et al. 2024; Pigg et al. 2010; Sachau et al. 2023; Vuilleumier et al. 2015) studies were included in this review. Studies that were excluded had either wrong outcome measure, wrong study population, wrong reliability estimates, or were abstracts only.

### 3.1 Study Characteristics

Of the included studies, 11 studies investigated TSP in healthy individuals (Cathcart et al. 2009; Costa et al. 2019, 2017; De Vita et al. 2022; Graven‐Nielsen et al. 2015; Izumi et al. 2022; Kong et al. 2013; Mailloux et al. 2021; Marcuzzi et al. 2017; Nothnagel et al. 2017; Pigg et al. 2010), seven studies assessed clinical population (Biurrun Manresa et al. 2011; Dams et al. 2022; de la Coba et al. 2018; de Oliveira et al. 2023; Othman et al. 2024; Sachau et al. 2023; Vuilleumier et al. 2015), and four assessed both healthy and clinical populations (Baad‐Hansen et al. 2015; Brady et al. 2023; Knox et al. 2024; Middlebrook et al. 2020). Most studies included men and women; only three studies included only women. A total of 1177 participants (age range: 19 to 78) are included across all studies, of which 50.0% (*n* = 589) are healthy participants and 43.2% (*n* = 508) males. The clinical population includes rheumatoid arthritis, low back pain, fibromyalgia, musculoskeletal trauma and atypical odontalgia (Table 1).

**TABLE 1:** Study's demographics.

Study	*n*	Male (*n*)		Female (*n*)		Overall	Age (years)	
		Healthy	Clinical	Healthy	Clinical		Male	Female
Baad‐Hansen et al. (2015)	113							
	H: 68	26	—	42	—	42 ± 15	—	—
	AO: 45	—	7	—	38	56 ± 13	—	—
Biurrun Manresa et al. (2011)	25	—	13	—	12	51 (23–78)	—	—
Brady et al. (2023)	88							
	H1: 20	10	—	10	—	26 (23–32)	—	—
	H2: 25	10	—	15	—	31 (28–46)	—	—
	RA: 18	—	5	—	13	58 (55–65)	—	—
	LBP: 25	—	8	—	17	57 (48–65)	—	—
Cathcart et al. (2009)	20	9	—	11	—	—	27 ± 6.4	23 ± 3.6
Costa et al. (2017)	28	—	—	28	—	—	—	21.6 ± 1.81
Costa et al. (2019)	40	20	—	20		—	26.0 ± 4.5	24.6 ± 3.9
Dams et al. (2022)	30	—	—	—	30	—	—	57 ± 10
de la Coba et al. (2018)	65							
	FM: 35	—	—	—	35	—	—	53.11 ± 8.28
	RA: 30	—	—	—	30	—	—	53.07 ± 10.55
de Oliveira et al. (2023)	28	—	11	—	17	33.9 ± 11.9	—	—
De Vita et al. (2022)	33	13	—	20	—	19 ± 1.3	—	—
Graven‐Nielsen et al. (2015)	136	68	—	68	—	—	31.7 ± 13.7	31.7 ± 14.0
Izumi et al. (2022)	21	11	—	10	—	27 (20–38)	—	—
Knox et al. (2024)	75							
	Y: 25	12	—	13	—	25.2 ± 3.6	—	—
	O: 25	12	—	13	—	71.4 ± 7.8	—	—
	OP: 25	—	12	—	13	69.4 ± 6.2	—	—
Kong et al. (2013)	19	10	—	9	—	—	29.7 ± 10.9	28.2 ± 10.9
Mailloux et al. (2021)	24	12	—	12	—	28.3 ± 11.0	—	—
Marcuzzi et al. (2017)	42	21	—	21	—	30.2 ± 10	—	—
Middlebrook et al. (2020)	40							
	H: 20	10	—	10	—	27.55 ± 8.06	—	—
	T: 20	—	14	—	6	44.8 ± 19.32	—	
Nothnagel et al. (2017)	22	10	—	12	—	46.6 ± 13.0	38.2 ± 13.1	54.2 ± 6.8
Othman et al. (2024)	47	—	40	—	7	25.19 ± 8.96	—	—
Pigg et al. (2010)	21	8	—	13	—	40.4 (24–71)	39.8 (24–71)	40.8 (26–54)
Sachau et al. (2023)	60	—	34	—	26	58.0 ± 15.3	—	—
Vuilleumier et al. (2015)	89	—	49	—	50	56 ± 15.9	—	—

Abbreviations: AO, atypical odontalgia; FM, fibromyalgia; H (1 or 2), healthy participant, 1 or 2 depicts subgroups of healthy participants; LBP, low back pain; *n*, sample size; O, older participants; OP, older participants with low back pain; RA, rheumatoid arthritis; T, musculoskeletal trauma patients; Y, younger participants.

The majority of studies reported TSP reliability across two visits (*n* = 17, 77.3%), two studies across three visits (9.1%), and three studies assessed reliability within one visit (13.6%). Within‐session time intervals range between 2 and 180 min, with five studies not reporting the time interval between sessions, and between‐session time intervals range between 2 days and 16 weeks (Table 2).

**TABLE 2:** Study's methodological description: number of sessions, intervals between sessions and reliability measurements.

Study	Visits	Sessions	Within‐sessions	Between‐sessions		Reliability measures	ICC model
			min	Days	Week		
Baad‐Hansen et al. (2015)	2	2	n.r.		1	ICC, CV	n.r.
Biurrun Manresa et al. (2011)	3	1		7.7 ± 2.6		ICC, CV, B‐A LoA	ICC (3, 1) absolute agreement
Brady et al. (2023)	2	1	n.r.		1–3	ICC, B‐A LoA	ICC (2, 1) absolute agreement
Cathcart et al. (2009)	1	1	60			ICC, CR	n.r.
Costa et al. (2017)	2	1			1	ICC, CV	n.r.
Costa et al. (2019)	2	1			1	ICC, SEM	ICC (2, 1) absolute agreement
Dams et al. (2022)	2	2	60		1	ICC, SEM, B‐A LoA	ICC (3, 1), ICC (2, 1), absolute agreement
de la Coba et al. (2018)	2	1		4–7		ICC	n.r.
de Oliveira et al. (2023)	1	1	20			ICC, CV	ICC (2, k) absolute agreement
De Vita et al. (2022)	2	1		7		ICC, B‐A LoA	ICC (3, 2) absolute agreement
Graven‐Nielsen et al. (2015)	2	1		7–21		ICC, CV, B‐A LoA	ICC (3, 1) consistency
Izumi et al. (2022)	2	2	n.r.		1	ICC	ICC (1, k), ICC (3, k)
Knox et al. (2024)	2	2	n.r.	2–7		ICC	ICC (3, k) absolute agreement
Kong et al. (2013)	2	1	2–30	7		ICC, B‐A LoA	ICC (3, 10) absolute agreement
Mailloux et al. (2021)	1	1	20			ICC, CV	ICC (2, k) absolute agreement
Marcuzzi et al. (2017)	3	1			8, 16	ICC, SEM	ICC (3, 1) consistency
Middlebrook et al. (2020)	2	2	> 2	2–7		ICC	ICC (3, k) absolute agreement
Nothnagel et al. (2017)	2	1			10.0 ± 2.9	ICC, B‐A LoA, SEM	ICC (2, k) absolute agreement
Othman et al. (2024)	2	1			1	ICC, SEM	ICC (3, 1) consistency
Pigg et al. (2010)	2	2	n.r.		1–3	ICC	NR
Sachau et al. (2023)	2	3	180		3	ICC	ICC (2, k)
Vuilleumier et al. (2015)	2	1		7–28		ICC, CV, CR, SEM	ICC (3, k) absolute agreement

Abbreviations: B‐A LoA, Bland–Altman Limits of Agreement; CR, coefficient of repeatability; CR, coefficient of residuals; CV, coefficient of variation; ICC, intraclass correlation coefficient; n.r., not reported; SEM, standard error of measurement; Session, total number of sessions per study.

Variations in TSP paradigms are presented in Table 3. A total of three stimulus types, mechanical (82.6%, *n* = 19), thermal (8.7%, *n* = 2) and electrical (8.7%, *n* = 2), were used to assess TSP. Mechanical stimuli were subcategorised based on contact area: small contact area (*n* = 16) (e.g., pinprick, monofilament), medium contact area (*n* = 2) (e.g., pressure algometer) and large contact area (*n* = 1) (e.g., cuff pressure). For thermal stimulation, a thermal probe was used, and for electrical stimulation, a constant current stimulator was used.

**TABLE 3:** Study's temporal summation of pain paradigm.

Study	Test paradigm				Site	TSP calculation
	Type	Intensity	No. of repeated stimuli	ISI		
Baad‐Hansen et al. (2015)	Pinprick – Mechanical – Small contact area	Fixed – 128 mN	10	1/s	– Buccal gingiva of painful tooth – Gingia adjacent to tooth 23–24 – Thenar eminence of right hand	Mean pain rating for 10‐stimuli divided by mean pain rating for single stimulus
Biurrun Manresa et al. (2011)	Electrical	Individualised – Electrical current increase from 1 Ma in steps of 1 mA until a reflex was detected and a pain sensation was evoked	5	2 Hz	– Lateral malleolus, sural nerve	n.a.
Brady et al. (2023)	Pinprick – Mechanical – Small contact area	Fixed – 256 mN	10	1/s	– Rectus femoris – Brachioradialis	– WUD: Pain rating of single stimulus subtracted from average pain experienced during 10‐ stimuli – WUR: Average pain during 10‐stimuli divided by pain rating of single stimulus
Cathcart et al. (2009)	Pressure – Mechanical – Medium contact area	Individualised – Pressure pain detection threshold, pressure increase at 1 kg/s till first sensation of pain	10	1/s	– Dorsal surface of middle finger	10th minus first algometer pulse rating
Costa et al. (2017)	Pinprick – Mechanical – Small contact area	Individualised – Set of monofilaments with force rating between 0.008 g/mm^2^ to 300 g/mm^2^ were used to detect first sensation of pain	10	1/s	– Anterior temporalis – Masseter – TMJ	Mean rating of series stimulus divided by mean rating of single stimuli
Costa et al. (2019)	Pinprick – Mechanical – Small contact area	Individualised – Set of monofilaments with force rating between 0.008 g mm‐2 to 300 g mm‐2 were used to detect first sensation of pain	10	1 Hz	– Masseter – TMJ	Mean rating of series stimulus divided by mean rating of single stimuli
Dams et al. (2022)	Pinprick – Mechanical – Small contact area	Fixed – 256 mN	30	1/s	– Pectoral region	Difference between pain rating after train of pinprick stimuli and pain rating after first stimulation
de la Coba et al. (2018)	Monofilament – Mechanical – Small contact area	Fixed – 300 g	10	1 Hz	– Thenar eminence	Difference in pain rating between the 10th and first stimulus
de Oliveira et al. (2023)	Pinprick – Mechanical – Small contact area	Fixed – 256 mN	10	1 Hz	– L4/L5 joint line – Hand dorsum	Difference between highest pain intensity and pain level after a single stimulus
De Vita et al. (2022)	Thermal	Individualised – Temperature increase 2oC/s until suprathreshold^a^ value	Constant for 120 s	—	– Volar surface of forearm	n.a.
Graven‐Nielsen et al. (2015)	Cuff pressure – Mechanical – Large contact area	Individualised – Pre‐determined cuff pressure pain tolerance	10	1/s	– Lower leg – Upper arm	Ratio between average pain rating of 8–10th stimulus and 1st–3rd stimulus
Izumi et al. (2022)	Pinprick – Mechanical – Small contact area	Fixed – 60 g	10	1/s	– Tibialis anterior – Dorsum of hand	Difference in pain rating between the first and last stimuli
Knox et al. (2024)	Monofilament – Mechanical – Small contact area	Fixed – 300 g	10	1 Hz	– Metatarsal head of foot	Change score between pain ratings during repeated contacts and the first contact
Kong et al. (2013)	Thermal	Individualised – Stimulus temperature set at VAS of between 30 to 70	10	0.5 Hz	– Thenar eminence	Difference between peak pain score and pain response to the first pulse
Mailloux et al. (2021)	Pinprick – Mechanical – Small contact area	Fixed – 256 mN	10	1 Hz	– L4/L5 interspinous process – Hand dorsum	Difference between the highest NRS pain during the 10 repeated stimulus and the NRS pain after a single stimulus
Marcuzzi et al. (2017)	Pinprick – Mechanical – Small contact area	Fixed – 256 mN	10	1/s	– Dorsum of hand – Lower back, lateral to spinous process	Mean pain rating of 5 series of repeated pinprick stimuli divided by mean pain rating of 5 single stimuli
Middlebrook et al. (2020)	Pressure – Mechanical – Medium contact area	Individualised – First sensation of pain from PPT testing	10	1/s	– Extensor carpi radialis – Tibialis anterior – Lumbar erector spinae	Mean pain rating for pulse 1 to 4 (M1), 5 to 7 (M2) and 8 to 10 (M3) calculated. Ratio between M3 and M1 calculated.
Nothnagel et al. (2017)	Pinprick – Mechanical – Small contact area	Fixed – 256 mN	10	1 Hz	– Paraspinal lumbar area – Dorsum of hand	Mean pain rating of series divided by mean pain rating of single stimulus
Othman et al. (2024)	Pinprick – Mechanical – Small contact area	Fixed – 300 g	10	1 Hz	– Dorsum of distal forearm – Mid‐deltoid muscle	Difference between mean pain ratings of 10 series and mean pain rating of single stimuli
Pigg et al. (2010)	Pinprick – Mechanical – Small contact area	Individualised – Set of 7 pinprick between 8 to 512 mN – Pre‐determined force perceived as ‘slightly painful’	10	1/s	– Right cheek – Tip of tongue – Bilateral gingival mucosa of upper premolar region	Mean pain rating of repeated stimulus divided by mean pain rating of single stimuli
Sachau et al. (2023)	Neuropen with a Neurotip – Mechanical – Small contact area	Fixed – 40 g	10	1/s	– Area of body experiencing most pain – Contralateral area without pain	WUR ratio was calculated as ratio of last few stimuli of the row of ten divided by the single stimulus pain intensity
	0.7 mm CMS hair – Mechanical – Small contact area	Fixed – 10 g	10	1/s	– Area of body experiencing most pain – Contralateral area without pain	WUR ratio was calculated as ratio of last few stimuli of the row of ten divided by the single stimulus pain intensity
Vuilleumier et al. (2015)	Electrical	Individualised – Electrical current increased from 1 mA in steps of 0.5 mA until pain sensation was evoked	5	2 Hz	– Lateral malleolus, sural nerve	n.a.

Abbreviations: mN, millinewton; n.a., not assessed; n.r., not reported; PPT, pain pressure threshold; VAS, visual analog scale; WUD, wind‐up difference; WUR, wind‐up ratio.

^a^ Suprathreshold value defined as average of threshold and tolerance temperature.

The intensity of stimulus was categorised into either ‘fixed’ or ‘individualised’. ‘Fixed’ stimulus intensity is defined as having all participants receiving an identical stimulation intensity (e.g., all participant received the pinprick 256 mN stimulus). ‘Individualised’ stimulus intensity is defined as each participant receiving a stimulation intensity based on their individual subjective threshold (e.g., thermal probe set at a temperature where the participant reports a 7/10 pain rating). The majority of studies using a small contact mechanical stimulus utilised ‘fixed’ intensity (*n* = 13), and the remaining studies utilised‘individualised’ intensity (*n* = 3). All studies using medium and large contact mechanical stimuli utilised ‘individualised’ intensities. In all studies using electrical and thermal stimuli, the intensity was ‘individualised’.

Most studies (*n* = 18) used 10 identical repeated stimuli to assess a temporal response, two studies had 5 repeated stimuli, one study had 30 repeated stimuli, and one study applied a constant thermal stimulation for 120 s. Except for the study reporting a constant stimulation, the ISI was similar across studies, with most studies using 1 stimulus per second (1 Hz frequency), and two studies using a slightly different frequencies (2 Hz and 0.5 Hz).

Various body sites were tested across paradigms (Table 3). Test sites are conveniently categorised into eight broad categories: low back (*n* = 5), hand (*n* = 9), ankle/ft region (*n* = 3), oral region (i.e., gingiva and tongue) (*n* = 2), leg (i.e., thigh and leg region) (*n* = 4), arm (i.e., upper arm and forearm region) (*n* = 5), mandibular region (i.e., cheeks, temporomandibular joint, temporalis) (*n* = 3) and pectoral/deltoid region (*n* = 2).

### 3.2 Reliability of TSP

Across all included studies, a total of 137 ICC estimates were reported (Table S1). All studies reported within‐session and between‐session reliability, except five studies only assessed within‐session reliability, and seven assessed only between‐session reliability. Overall, the ICC ranged from −0.31 to 0.97. Reliability estimates for TSP were grouped into within‐session and between‐session in healthy, clinical and mixed populations (mixed being a combination of clinical and healthy populations).

#### 3.2.1 Healthy Population

Across the 14 studies that reported reliability for a healthy population, 70 ICC estimates were reported, ranging from −0.31 to 0.97 (Table S1). Grouping ICCs into reliability classifications, there were four (5.7%) reporting excellent, 13 (18.6%) good, 20 (28.6%) moderate and 33 (47.1%) of poor reliability. Forty ICC estimates reported on between‐sessions, and 33 were from within‐sessions. Meta‐analysis for between‐session and within‐session reliability in healthy individuals was conducted and reported below.

##### 3.2.1.1 Between‐Session Reliability

Meta‐analysis found between‐session reliability was poor with high heterogeneity (ICC = 0.49, 95% CI: 0.40–0.58, *I* ^2^ = 81.43%, 35 estimates, 1208 subjects) (Figure S1). Subgroup analysis, based on stimulus type, found that the reliability of mechanical stimulus was poor with high heterogeneity (ICC = 0.47, 95% CI: 0.38–0.56, *I* ^2^ = 80.20%), and the reliability of thermal stimulus was good (ICC = 0.78, 95% CI: 0.66–0.90, *I* ^2^ = 9.29%).

**FIGURE 2:**
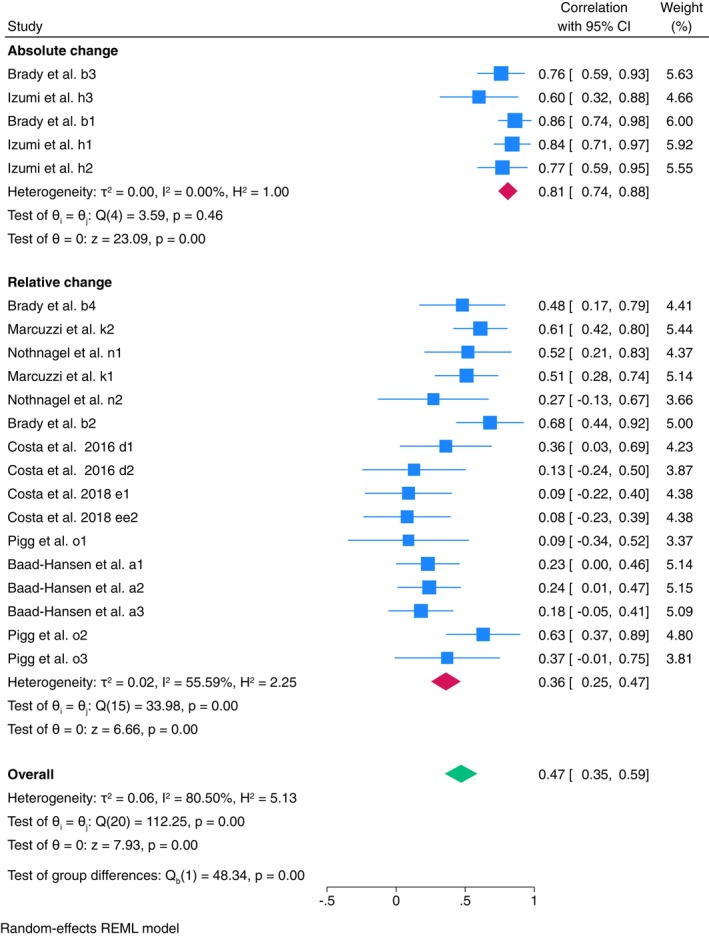
Forest plot of meta‐analysis of ‘small contact area’ stimulus type in TSP between‐session reliability in healthy population, with subgroup analysis of ‘absolute change’ vs. ‘relative change’ calculation methods.

Further meta‐analysis was conducted for mechanical stimulus (Figure S2). Large contact area stimulus had moderate reliability (ICC = 0.52, 95% CI: 0.35–0.69, *I* ^2^ = 72.47%), medium contact area had poor reliability (ICC = 0.44, 95% CI: 0.26–0.63, *I* ^2^ = 71.98%), and small contact area had poor reliability (ICC = 0.47, 95% CI: 0.35–0.59, *I* ^2^ = 80.50%). Reliability of small contact area stimulus was further analysed due to the larger number of estimates reported. Meta‐analysis shows good reliability when the absolute change was utilised (ICC = 0.81, 95% CI: 0.74–0.88, *I* ^2^ = 0.00%), and poor reliability when utilising relative change (ICC = 0.36, 95% CI: 0.25–0.47, *I* ^2^ = 55.59%) (Figure 2). To investigate the heterogeneity for ‘relative change’, a univariate meta‐regression was conducted. Results indicate that body test site is a significant predictor (*β* = −0.09, 95% CI: −0.14–0.03, *p* < 0.01) of the correlation, with the between‐study variance (*R* ^2^ = 100%) indicating that body test site fully accounts for the heterogeneity observed across studies.

##### 3.2.1.2 Within‐Session Reliability

**FIGURE 3:**
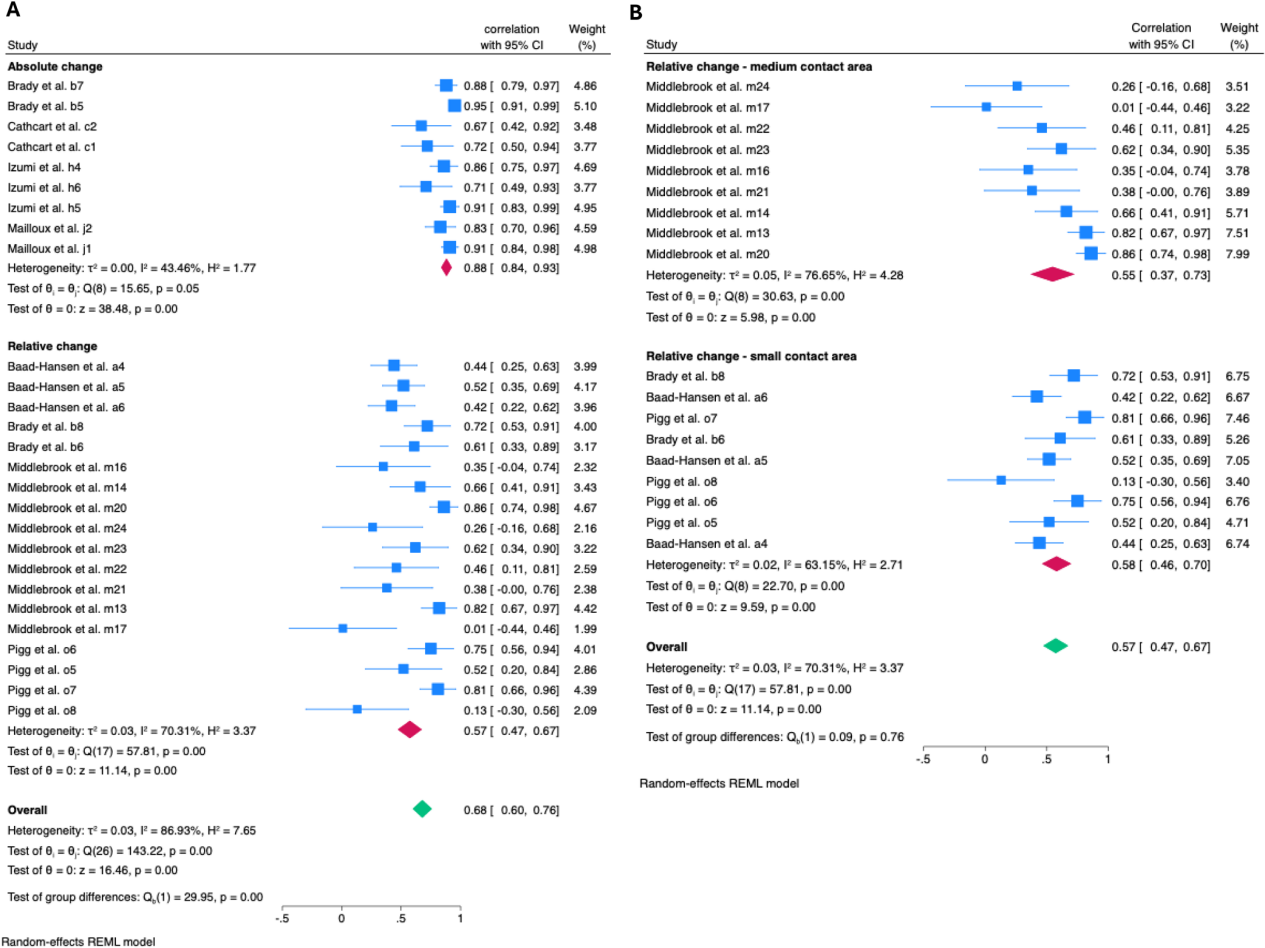
Forest plot of meta‐analysis of within‐session reliability in healthy population: (A) Subgroup analysis of calculation method reliability in healthy population when mechanical stimulus was applied; (B) Subgroup analysis of stimulus type reliability when using ‘relative change’ calculation method in different type of mechanical stimulus.

Meta‐analysis for within‐session reliability found moderate reliability with high heterogeneity (ICC = 0.69, 95% CI: 0.61–0.77, *I* ^2^ = 91.69%, 28 estimates, 728 subjects) (Figure S3). Subgroup analysis shows that the reliability of mechanical stimulus was moderate (ICC: 0.68, 95% CI: 0.60, 0.76, *I* ^2^ = 86.93%, 27 estimates, 728 participants). Further analysis of mechanical stimulus was undertaken by comparing absolute and relative change calculation methods, indicating that absolute change had good reliability (ICC = 0.88, 95% CI: 0.84–0.93, *I* ^2^ = 43.46%). The relative change method was found to be less reliable, but still classified as good (ICC = 0.57, 95% CI: 0.47–0.67, *I* ^2^ = 70.31%) (Figure 3A). Further subgroup analysis within relative change calculation found moderate reliability for both ‘medium contact area’ (ICC = 0.55, 95% CI: 0.37–0.73, *I* ^2^ = 76.65%) and ‘small contact area’ applications (ICC = 0.58, 95% CI: 0.46–0.70, *I* ^2^ = 63.15%) (Figure 3B). Univariate meta‐regression was conducted for both types of stimuli. For the ‘medium contact area’ stimulus, results indicate that body test site was a significant predictor (*β* = −0.1370, *p* < 0.01) of the correlation, explaining most of the between‐study variance (*R* ^2^ = 71.73%), with moderate residual heterogeneity (*I* ^2^ = 48.20%). However, for the ‘small contact area’ stimulus, the body test site was not a significant moderator (*β* = −0.06, *p* = 0.25), explaining only little of between‐study variance (*R* ^2^ = 21.66%) with moderate residual heterogeneity (*I* ^2^ = 47.60%).

#### 3.2.2 Clinical Population

Of the eight studies that reported TSP reliability for a clinical population, 24 ICC estimates were reported, ranging from −0.002 to 0.90 (Table S1). ICCs were grouped according to classification: four (16.7%) of good, 13 (54.2%) moderate and seven (29.2%) of poor reliability. Fourteen ICC estimates reported on between‐session calculations, and 10 were from within‐sessions.

##### 3.2.2.1 Between‐Session Reliability

**FIGURE 4:**
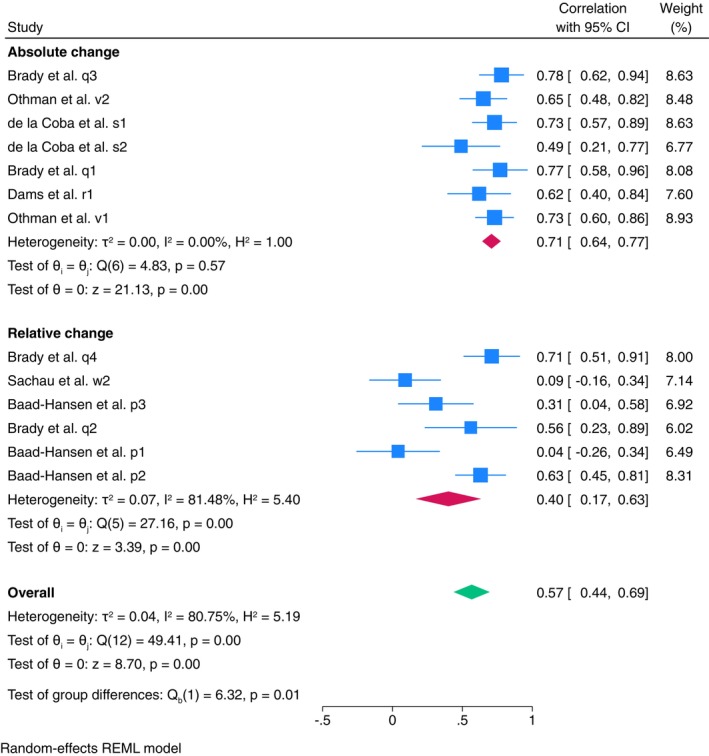
Forest plot of meta‐analysis of between‐session reliability of clinical population with subgroup analysis of calculation methods.

Meta‐analysis for between‐session reliability showed moderate reliability (ICC = 0.57, 95% CI: 0.44–0.69, *I* ^2^ = 80.75%, 13 estimates, 470 subjects) (Figure 4). Subgroup meta‐analysis, based on calculation method (i.e., absolute and relative change), indicated that when the absolute change was used, there was moderate reliability with no heterogeneity (ICC = 0.71, 95% CI: 0.64–0.77, *I* ^2^ = 0.00%). However, there was poor reliability and high heterogeneity when relative change was applied (ICC = 0.40, 95% CI: 0.17–0.63, *I* ^2^ = 81.48%). Univariate meta‐regression was conducted with body test site as a predictor; however, results did not show a significant association (*R* ^2^ = 0%, *p* = 0.74), and 81.2% residual heterogeneity. Stimulus type was not used as a predictor, as all studies utilised a small contact area stimulus.

##### 3.2.2.2 Within‐Session Reliability

**FIGURE 5:**
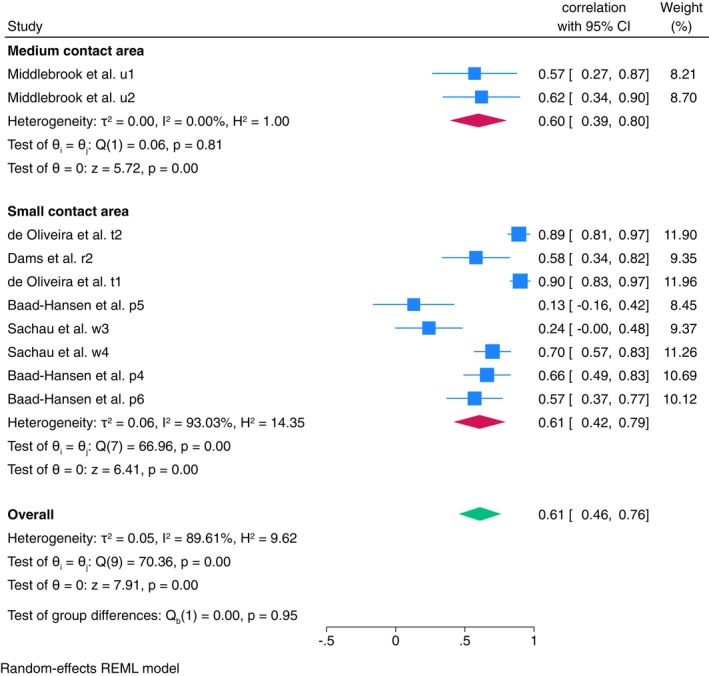
Forest plot of meta‐analysis of within‐session reliability of clinical population with subgroup analysis of mechanical stimulus type.

Meta‐analysis for within‐session reliability showed moderate reliability (ICC = 0.61, 95% CI: 0.46–0.76, *I* ^2^ = 89.61%, 10 estimates, 381 subjects) (Figure 5). Subgroup analysis for the ‘medium contact area’ had moderate reliability with no heterogeneity (ICC = 0.60, 95% CI: 0.39–0.80, *I* ^2^ = 0.00%), whereas ‘small contact area’ had moderate reliability (ICC = 0.61, 95% CI: 0.42–0.79, *I* ^2^ = 93.03%). Univariate meta‐regression indicates that the calculation method is a significant predictor (coefficient: −0.65, *p* = 0.02) of the correlation, explaining almost half of the between‐study variance (*R* ^2^ = 44.12%). However, there was high residual heterogeneity (*I* ^2^ = 81.79%).

#### 3.2.3 Mixed Population

Only one study reported ICC estimates of both healthy and clinical populations combined (Table S1). The between‐session ICC estimate was good (ICC = 0.83), and within‐session reliability was moderate (Session A, ICC = 0.74) and good (Session B, 0.90). Meta‐analysis was not conducted as there was only one study.

### 3.3 Reliability of a Single Stimulus and Repeated Stimuli

The reliability of single‐stimulus and repeated‐stimulus paradigms was assessed by four studies (Table S2). All four studies reported on clinical populations and a total of 38 ICC estimates were reported, with 19 ICC estimates for single‐stimulus and repeated‐stimulus paradigms each. ICC estimates ranged from 0.69 to 0.99 for the single stimulus; five (26.3%) demonstrated moderate reliability, six (31.6%) good reliability and eight (42.1%) excellent reliability. The repeated stimulus had ICC estimates ranging from 0.62 to 0.99; 8 (42.1%) were of excellent reliability, 9 (47.4%) good reliability and 2 (10.5%) of moderate reliability.

**FIGURE 6:**
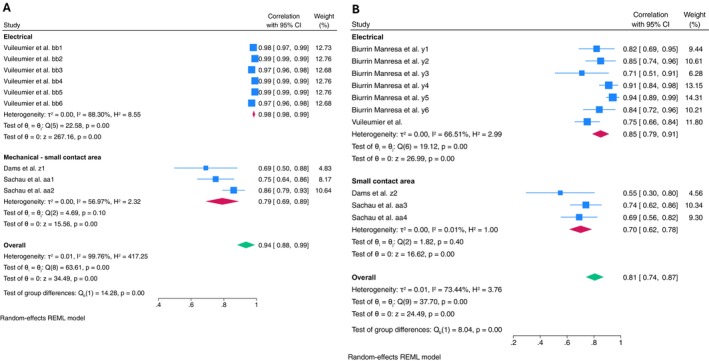
Forest plot of meta‐analysis of single‐stimulus reliability: (A) within‐session reliability with subgroup analysis of stimulus type, (B) between‐session reliability with subgroup analysis of stimulus.

Meta‐analysis of single‐stimulus within‐session reliability found excellent reliability with high heterogeneity (ICC = 0.94, 95% CI: 0.88–0.99, *I* ^2^ = 99.76%, 9 estimates, 684 subjects). Subgroup analysis of stimulus type indicated that electrical stimulus had excellent reliability with high heterogeneity (ICC = 0.98, 95% CI: 0.98–0.99, *I* ^2^ = 88.30%), and ‘small contact area’ stimulus had good reliability with moderate‐high heterogeneity (ICC = 0.79, 95% CI: 0.69–0.89, *I* ^2^ = 56.97%) (Figure 6A).

Meta‐analysis of single‐stimulus between‐session reliability had good reliability (ICC = 0.81, 95% CI: 0.74–0.87, *I* ^2^ = 73.44%). Subgroup analysis showed good reliability with high heterogeneity when an electrical stimulus was applied (ICC = 0.85, 95% CI: 0.79–0.91, *I* ^2^ = 66.51%) and moderate reliability with almost no heterogeneity when a mechanical stimulus was applied (ICC = 0.70, 95% CI: 0.62–0.78, *I* ^2^ = 0.01%) (only ‘small contact area’ stimulus was used) (Figure 6B).

Meta‐analysis of repeated‐stimulus within‐session reliability demonstrated excellent reliability with high heterogeneity (ICC = 0.97, 95% CI: 0.95–0.99, *I* ^2^ = 98.40%). Subgroup analysis indicated excellent reliability with moderate‐high heterogeneity when electrical stimulus was applied (ICC = 0.99, 95% CI: 0.98–0.99, *I* ^2^ = 69.39%), and excellent reliability with almost no heterogeneity when mechanical stimulus was applied (ICC = 0.91, 95% CI: 0.88–0.94, *I* ^2^ = 0.06%) (only ‘small contact area’ stimulus was used).

**FIGURE 7:**
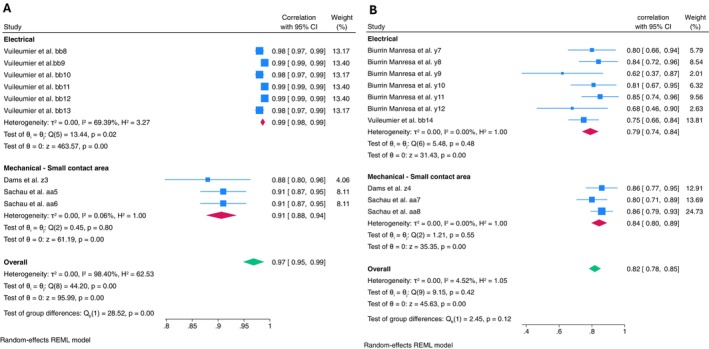
Forest plot of meta‐analysis of repeated‐stimulus reliability: (A) within‐session reliability with subgroup analysis of stimulus type; (B) between‐session reliability with subgroup analysis of stimulus type.

Meta‐analysis of repeated stimulus between session found good reliability with low heterogeneity (ICC = 0.82, 95% CI: 0.78–0.85, *I* ^2^ = 4.52%) (Figure 7). Subgroup analysis showed good reliability with no heterogeneity when an electrical stimulus was applied (ICC = 0.79, 95% CI: 0.74–0.84, *I* ^2^ = 0.00%), and good reliability with no heterogeneity when a mechanical stimulus was applied (ICC = 0.84, 95% CI: 0.80–0.89, *I* ^2^ = 0.00%) (only ‘small contact area’ stimulus was used).

### 3.4 Risk of Bias and Quality Assessment

Risk of bias and quality assessment are presented in Table 4. Thirteen studies were rated as ‘doubtful’ and nine studies were rated as ‘adequate’. Considering that single‐stimulus, repeated‐stimulus and TSP score (i.e., the absolute and relative change of single‐ and repeated‐stimulus) are continuous outcomes, COSMIN‐ROB standards 5–7 are not applicable. Using the QACMRR tool, all studies were found to have an overall positive rating, with three being rated ‘excellent’ and 18 as ‘very good’.

**TABLE 4:** Risk of bias and QACMRR.

Study	Psychometric properties reported	COSMIN‐ROB	COSMIN rating	QACMRR
Baad‐Hansen et al. (2015)	Reliability	Doubtful	−	Very good
Biurrun Manresa et al. (2011)	Reliability	Adequate	−	Excellent
Brady et al. (2023)	Reliability	Doubtful	−	Very good
Cathcart et al. (2009)	Reliability	Adequate	−	Very good
Costa et al. (2017)	Reliability	Adequate	−	Very good
Costa et al. (2019)	Reliability	Adequate	−	Very good
Dams et al. (2022)	Reliability	Adequate	−	Excellent
de la Coba et al. (2018)	Reliability	Doubtful	−	Very good
de Oliveira et al. (2023)	Reliability	Adequate	+	Excellent
De Vita et al. (2022)	Reliability	Doubtful	+	Very good
Graven‐Nielsen et al. (2015)	Reliability	Doubtful	−	Very good
Izumi et al. (2022)	Reliability	Doubtful	−	Very good
Knox et al. (2024)	Reliability	Doubtful	+	Very good
Kong et al. (2013)	Reliability	Adequate	+	Very good
Mailloux et al. (2021)	Reliability	Doubtful	+	Very good
Marcuzzi et al. (2017)	Reliability	Doubtful	−	Very good
Middlebrook et al. (2020)	Reliability	Adequate	−	Very good
Nothnagel et al. (2017)	Reliability	Adequate	−	Very good
Othman et al. (2024)	Reliability	Doubtful	−	Very good
Pigg et al. (2010)	Reliability	Doubtful	−	Very good
Sachau et al. (2023)	Reliability	Doubtful	−	Very good
Vuilleumier et al. (2015)	Reliability	Doubtful	+	Very good

*Note:* Criteria for good measurement properties: sufficient ‘+’; insufficient ‘−’; indeterminate ‘?’.

Abbreviations: COSMIN, Consensus‐based standards for the selection of health measurement instruments—risk of bias checklist; QACMRR, quality appraisal for clinical measurement research report evaluation form; ROB, risk of bias.

## 4 Discussion

The objective of this review was to determine if TSP is a reliable measure. A total of 22 studies, with 137 reliability estimates, were included in this review. Meta‐analysis showed high variability across studies, reporting poor to excellent reliability. Recent work highlighted that a large number of diverse methods are used to assess TSP, and that there is a lack of expert recommendations or a standardised methodology (Kielstra et al. 2024). Our work demonstrates that these large variations and the lack of methodological uniformity may explain the heterogeneity of reliability across studies.

TSP assesses the facilitatory pathway by the perception of increased pain intensity to repeated identical noxious stimulus (Arendt‐Nielsen et al. 2018). The TSP effect can be influenced by various factors, such as participant demographics (e.g., gender, age), and multiple methodological approaches (e.g., modalities, instruments, train characteristics, calculations, test sites) (Eckert et al. 2017). Across the studies included in this review, multiple modalities with varying instruments were used as the noxious stimulus, including mechanical, thermal and electrical stimulus. Within mechanical stimulus, ‘small contact area’ stimuli were most frequently utilised, specifically the pinprick stimulus. Despite the high frequency of usage, the reliability of the ‘small contact area’ stimulus remains poor to moderate in all analysed instances. The popularity of a pinprick stimulus might be attributed to the published protocol of quantitative sensory testing (QST) (Rolke et al. 2006), where TSP was assessed using pinprick, as well as the relative ease of usage, low cost of the instrumentation and its accessibility, making it feasible for laboratory and clinical settings (Kielstra et al. 2024). On the other hand, electrical and thermal stimuli were only assessed by three studies. The application of an electrical or thermal stimulus requires expensive equipment, which often necessitates training to operate the equipment, and has a higher risk of adverse events such as thermal burns. Ease of accessibility and complexity of usage may contribute to the infrequent use of thermal and electrical stimuli as the noxious stimulus to assess TSP.

From our review, absolute change had much stronger reliability compared to relative change. One major factor for the large difference in reliability could be due to the intensity of the initial stimulus. To elicit a wind‐up phenomenon in the dorsal horn of the spinal cord, the initial single‐stimulus should be nociceptive, but it is not necessary to elicit pain (Herrero et al. 2000). The lack of pain response to the initial stimulus is a common limitation of calculating TSP based on relative change, where participants reported zero on a NRS scale or ‘no pain’. In this case, as the TSP cannot be calculated (i.e., last stimulus pain rating cannot be divided by the ‘0’ pain rating of the initial stimulus), the participant's data would have to be excluded from the analysis. To mitigate this limitation, some studies utilised arbitrary or complex calculation formulas, such as applying a logarithmic function. However, such calculations alter the results and add complexity to the analysis and understanding of the results, making direct comparisons across studies difficult.

We found a total of over 20 body test sites used for the assessment of TSP. Notably, two studies used a participant's self‐reported painful area as a test site, alongside the contralateral non‐painful site. Analysis from this review indicates that body test site plays a significant factor in comparing the reliability of TSP across studies. In studies assessing participants with clinical conditions such as atypical odontalgia or low back pain, the test sites utilised were typically the area of pain and the contralateral site without pain, with some studies assessing an additional remote non‐painful site. However, in studies with a healthy population, there was no justification or rationale for the chosen body test sites. As the physiological basis of the TSP response is the activation of nociceptive C‐fibres (Herrero et al. 2000), and the density of C‐fibres varies across different body sites (e.g., gingiva vs. quadriceps) (Mancini et al. 2014), the physiological response at different body test sites should be an important factor for consideration.

A total of three different types of stimuli were identified: mechanical, thermal and electrical. Within mechanical stimuli, there are three sub‐groups based on the probe size having a small, medium or large contact area. The reliability varies between and within each stimulus group. Moderate to excellent reliability was found in electrical and thermal stimulation. However, as the number of studies and ICC estimates was relatively small, the confidence concerning its reliability remains low. Overall, mechanical stimulus had poor to moderate reliability, which was found to be influenced by the calculation method and the body test sites. Different types of stimuli (e.g., mechanical, thermal and electrical) were found to not impact TSP. A previous study found that pain rating increased by approximately 1.6 times between the first and last stimuli in the repeated‐stimulus paradigm, regardless of the type of stimulus (i.e., electrical, mechanical or thermal) in the healthy population (Herrero et al. 2000). However, such phenomenon has not been observed in clinical populations.

All studies in this review applied mechanical repeated stimuli at a frequency of 1 Hz. For electrical stimulation, frequency was set at 2 Hz, and thermal stimuli were applied at 0.5 Hz. The majority of studies applied 10 stimuli during the repeated‐stimulation phase, with only one study applying 30 stimuli, and two studies applying five stimuli. This range of frequency and number of stimuli is at odds with what was observed in a previous review, where results reported frequencies ranging from 0.03 to 200 Hz, and the number of stimuli ranging from two to 500 (Kielstra et al. 2024). The discrepancy between our review and the previous review (Kielstra et al. 2024) presents a concern as apparently a large number of methodologies utilised were not assessed for reliability. However, this could be due to studies analysing reliability as a secondary analysis and relevant results were not reported in the title and abstract, resulting in the study being excluded during the initial screening phase. This supports the notion that a large number of studies adopted a methodology based on convenience and availability of tools and the established practices within each research team (Kielstra et al. 2024). The absence of justification for a chosen methodology, especially without consideration for the reliability of the methodology, reduces the confidence of reported results.

Finally, it is possible that biological factors, such as the dynamic trait of endogenous pain modulation (Yarnitsky 2015), contribute to the weaker between‐session reliability compared to within‐session reliability in both healthy and clinical populations found in this review. This idea is supported by a previous meta‐analysis on the reliability of CPM, which found similar differences between within‐session and between‐session reliability, attributing the differential findings to the variability of endogenous inhibition over time (Nuwailati et al. 2022).

## 5 Strength and Limitations

To the best of our knowledge, this is the first meta‐analysis quantifying the reliability of TSP. The search strategy was kept broad to avoid missing potential studies, thereby increasing the confidence that relevant studies were included in this review. Data extraction and classification was kept consistent with the previously published review on CPM (Kennedy et al. 2016; Nuwailati et al. 2022) and TSP (Kielstra et al. 2024), thus providing consistent information to improve the quality of future reliability studies and potentially aid in future consensus recommendations for TSP assessments.

The quality of findings from meta‐analysis reported herein is limited by the quantity and quality of included studies. The high variability of TSP paradigms, participants' age and sex/gender, and different healthy and clinical populations across studies may contribute to the high heterogeneity found across meta‐analyses. Despite creating smaller subgroups for meta‐analysis, some residual heterogeneity remained unexplained. This warrants further investigation into additional moderators to better understand the sources of variation across studies.

## 6 Conclusion

This review suggests that TSP is a reliable measure, with its reliability highly dependent on the stimulus parameters, study methodology and population of interest. From this review, small contact area mechanical stimulus (e.g., pinprick) with absolute change calculation method, applied at a rate of 1 Hz, was found to be most reliable. However, the complex mechanisms modulating TSP need to be further explored, especially within the clinical population.

### 6.1 Recommendations for Future Research and Translation to Clinical Setting

Data reporting in TSP studies shares similar conundrums as CPM studies, where there is wide variability in data reported (Kennedy et al. 2016). Standardisation and consistency in data reporting will aid in the interpretation of results and comparison between studies, and enable strong recommendations for specific TSP paradigms for specific populations of interest. Considering the magnitude of change in TSP is of main interest, and the variability of methodology in calculating this change, recommendation of reporting absolute change and relative change (when appropriate for the paradigm chosen) should be included. Research involving healthy participants should also include collecting and reporting sociodemographic data, medical history and current health status (including mental health), pain coping strategies, motivation for participating in research, alcohol and drug abuse history, and frequency of any pain episodes during the last 3 to 6 months (Gierthmuhlen et al. 2015). These data will allow better interpretation and comparison with other research, improving quality of clinical research, and enabling results to be generalised to a population of interest (Gierthmuhlen et al. 2015; Kennedy et al. 2016).

While most TSP paradigms are found to have moderate to good reliability, consideration should also be given when translating experimental TSP testing to clinical routine assessment. Our results indicates that the ‘small contact area’ stimulus, in combination with absolute change calculation method, resulted in the highest reliability compared to other paradigm combinations in both healthy and clinical populations. Therefore, our results support a previous study conducted by the German Research Network on Neuropathic Pain (DFNS) which recommended calculation of the magnitude of a single pinprick and 10 consecutive pinprick stimuli of the same force (128 mN, when tested over face, and 256 mN, when tested over hand and foot) at an ISI rate of 1.0 Hz (Rolke et al. 2006). Future TSP reliability studies should provide reporting of better statistical design and analysis, such as providing a sample size calculation, appropriate reliability coefficient and 95% confidence interval, and measures of response stability. This will improve the validation of TSP as a robust prognostic factor in experimental and clinical pain studies.

## Conflicts of Interest

The authors declare no conflicts of interest.

## Supporting information


**Figure S1:** Forest plot of meta‐analysis for between‐session reliability in healthy population, with subgroup analysis of stimulus type (i.e., mechanical and thermal stimulus).

**Figure S2:** Forest plot of meta‐analysis of between‐session mechanical stimulus reliability in healthy population, with subgroup analysis of large, medium and small contact area mechanical stimulus.

**Figure S3:** Forest plot of meta‐analysis of within‐session reliability in healthy population, with subgroup analysis of stimulus type.

**Table S1:** ejp70097‐sup‐0004‐TableS1.docx.

**Table S2:** ejp70097‐sup‐0005‐TableS2.docx.

**Data S1:** ejp70097‐sup‐0006‐DataS1.docx.
