# Supplementary material for: Reliability of Temporal Summation of Pain in Healthy and Clinical Populations: A Systematic Review and Meta‐Analysis
Source: Eur J Pain. 2025 Aug 8;29(8):e70097. doi: 10.1002/ejp.70097 (PMC12333475; doi:10.1002/ejp.70097)
Supplement: Supplementary file 1 — Figure S1: Forest plot of meta‐analysis for between‐session reliability in healthy population, with subgroup analysis of stimulus type (i.e., mechanical and thermal stimulus). [file EJP-29-0-s003.docx]

**Figure S1**. Forest plot of meta-analysis for between-session reliability in healthy population, with subgroup analysis of stimulus type (i.e. mechanical and thermal stimulus).
