# Supplementary material for: Reliability of Temporal Summation of Pain in Healthy and Clinical Populations: A Systematic Review and Meta‐Analysis
Source: Eur J Pain. 2025 Aug 8;29(8):e70097. doi: 10.1002/ejp.70097 (PMC12333475; doi:10.1002/ejp.70097)
Supplement: Supplementary file 2 — Figure S2: Forest plot of meta‐analysis of between‐session mechanical stimulus reliability in healthy population, with subgroup analysis of large, medium and small contact area mechanical stimulus. [file EJP-29-0-s006.docx]

**Figure S2.** Forest plot of meta-analysis of between-session mechanical stimulus reliability in healthy population, with subgroup analysis of large, medium, and small contact area mechanical stimulus.
