# Supplementary material for: Reliability of Temporal Summation of Pain in Healthy and Clinical Populations: A Systematic Review and Meta‐Analysis
Source: Eur J Pain. 2025 Aug 8;29(8):e70097. doi: 10.1002/ejp.70097 (PMC12333475; doi:10.1002/ejp.70097)
Supplement: Supplementary file 3 — Figure S3: Forest plot of meta‐analysis of within‐session reliability in healthy population, with subgroup analysis of stimulus type. [file EJP-29-0-s002.docx]

**Figure S3.** Forest plot of meta-analysis of within-session reliability in healthy population, with subgroup analysis of stimulus type.
