# Supplementary material for: Reliability of Temporal Summation of Pain in Healthy and Clinical Populations: A Systematic Review and Meta‐Analysis
Source: Eur J Pain. 2025 Aug 8;29(8):e70097. doi: 10.1002/ejp.70097 (PMC12333475; doi:10.1002/ejp.70097)
Supplement: Supplementary file 4 — Table S1: ejp70097‐sup‐0004‐TableS1.docx. [file EJP-29-0-s004.docx]

**Table S1**. Temporal Summation of Pain reliability results in healthy population

|  | **between-session** |  | **within-session** |  |
| --- | --- | --- | --- | --- |
| **Study** | **ICC** | **Response stability** | **ICC** | **Response stability** |
| **Healthy population** |  |  |  |  |
| Baad-Hansen et al. [2] | GP: 0.23 ^a1^ | - | GP: 0.44 ^a4^ | CV: 36.6±30.6 |
|  | GNP: 0.24 ^a2^ | - | GNP: 0.52 ^a5^ |  |
|  | Hand: 0.18 ^a3^ | - | Hand: 0.42 ^a6^ |  |
| Brady et al. [4] | WUD: 0.86 (0.68, 0.94) ^b1^ | LoA: -0.12 (-1.53, 1.28) | WUD: 0.95 (0.87, 0.98) ^b5^ | LoA: 0.09 (-1.19, 1.36) |
|  | WUR: 0.68 (0.33, 0.86) ^b2^ | LoA: 1.08 (-6.25, 8.42) | WUR: 0.61 (0.26, 0.82) ^b6^ | LoA: -1.87 (-10.16, 6.42) |
|  | WUD: 0.76 (0.52, 0.89) ^b3^ | LoA: 0.15 (-1.23, 1.52) | WUD: 0.88 (0.74, 0.94) ^b7^ | LoA: -0.17 (-1.47, 1.12) |
|  | WUR: 0.48 (0.11, 0.73) ^b4^ | LoA: -1.36 (-9.33, 6.60) | WUR: 0.72 (0.41, 0.87) ^b8^ | LoA: -0.85 (-6.22, 4.52) |
| Cathcart et al. [5] | - | - | Finger: 0.72 ^c1^ | CR: 0.2 |
|  |  |  | Shoulder: 0.67 ^c2^ | CR: 0.28 |
| Costa et al. 2017 [7] | AT: 0.36 (-0.13, 0.71) ^d1^ | CV: 25.1% | - | - |
|  | Masseter: 0.13 (-0.38, 0.58) ^d2^ | CV: 28.7% |  |  |
|  | TMJ: -0.03 (-0.52, 0.45) ^d3^ | CV: 30.7% |  |  |
| Costa et al. 2019 [6] | TMJ: 0.09 (-0.19 – 0.37) ^e1^ | SEM: 0.86 | - | - |
|  | Masseter: 0.08 (-0.19 – 0.36) ^e2^ | SEM: 0.98 |  |  |
| de Vita et al. [11] | 0.71 (0.43, 0.86) ^f1^ | LoA: -0.38 (-3.11, 2.36) | - | - |
| Graven-Nielsen et al. [16] | Leg: 0.60 (0.43, 0.72) ^g1^ | LoA: 0.09 (-0.6, 1.2)  CV: 32.0% | - | - |
|  | Arm: 0.43 (0.19, 0.59) ^g2^ | LoA: 0.02 (-1.9 – 2.0)  CV: 22.6% | - | - |
| Izumi et al. [18] | TA: 0.84 (0.61, 0.93) ^h1^ | - | TA: 0.86 (0.64, 0.94) ^h4^ | - |
|  | Deltoid: 0.77 (0.43, 0.90) ^h2^ | - | Deltoid: 0.91 (0.78, 0.96) ^h5^ | - |
|  | Hand:0.60 (0.13, 0.83) ^h3^ | - | Hand: 0.71 (0.28, 0.88) ^h6^ | - |
| Kong et al. [22] | 0.83 (0.58, 0.93) ^i1^ | - | 0.97 (0.94, 0.99) ^i2^ | LoA: -5.1 (-10.1 – -0.03) |
| Mailloux et al. [24] | - | - | Hand: 0.91 (0.79 – 0.96) ^j1^ | CV: 60.3% |
|  |  |  | Back: 0.83 (0.59 – 0.93) ^j2^ | CV: 74.5% |
| Marcuzzi et al. [26] | Hand: 0.51 (0.32, 0.68) ^k1^ |  |  | SEM: 1.3 |
|  | Back: 0.61 (0.44, 0.75) ^k2^ |  |  | SEM: 1.2 |
| Middlebrook et al. [28] | Rater 1 – |  | Day 1 – |  |
|  | ULR: -0.31 (-2.67 – 0.52) ^m1^ | SEM, Day 1: 7.13, Day 2: 3.77 | ULR: 0.82 (0.53 – 0.93) ^m13^ | - |
|  | ULL: 0.55 (-0.22 – 0.84) ^m2^ | SEM, Day 1: 7.35, Day 2: 6.45 | ULL: 0.66 (0.15 – 0.86) ^m14^ | - |
|  | LLR: -0.04 (-1.73 – 0.60) ^m3^ | SEM, Day 1: 9.91, Day 2: 9.00 | LLR: -0.17 (-2.23 – 0.56) ^m15^ | - |
|  | LLL: 0.27 (-0.69 – 0.70) ^m4^ | SEM, Day 1: 7.11, Day 2: 5.26 | LLL: 0.35 (-0.48 – 0.73) ^m16^ | - |
|  | SR: 0.61 (-0.09 – 0.86) ^m5^ | SEM, Day 1: 6.03, Day 2: 5.42 | SR: 0.01 (-1.59 – 0.62) ^m17^ | - |
|  | SL: 0.10 (-1.44 – 0.65) ^m6^ | SEM, Day 1: 5.15, Day 2: 5.87 | SL: -0.03 (-1.63 – 0.59) ^m18^ | - |
|  | Rater 2 – |  | Day 2 – |  |
|  | ULR: 0.32 (-0.94 – 0.75) ^m7^ | SEM, Day 1: 6.64, Day 2: 4.15 | ULR: -0.02 (-1.33 – 0.58) ^m19^ | - |
|  | ULL: 0.56 (-0.27 – 0.85) ^m8^ | SEM, Day 1: 4.20, Day 2: 5.65 | ULL: 0.86 (0.59 – 0.95) ^m20^ | - |
|  | LLR: 0.23 (-1.09 – 0.71) ^m9^ | SEM, Day 1: 9.11, Day 2: 7.82 | LLR: 0.38 (-0.66 – 0.76) ^m21^ | - |
|  | LLL: 0.41 (-0.26 – 0.75) ^m10^ | SEM, Day 1: 9.14, Day 2: 5.56 | LLL: 0.46 (-0.18 – 0.77) ^m22^ | - |
|  | SR: 0.01 (-1.72 – 0.63) ^m11^ | SEM, Day 1: 6.77, Day 2: 6.87 | SR: 0.62 (-0.03 – 0.85) ^m23^ | - |
|  | SL: 0.89 (0.72 – 0.96) ^m12^ | SEM, Day 1: 5.71, Day 2: 5.80 | SL: 0.26 (-0.85 – 0.71) ^m24^ | - |
| Nothnagel et al. [30] | Back: 0.52 (0.13, 0.77) ^n1^ | LoA: 0.02 (-0.43, 0.46)  SEM: 0.16 | - | - |
|  | Hand: 0.27 (-0.18, 0.62) ^n2^ | LoA: -0.01 (-0.52, 0.51)  SEM: 0.18 | - | - |
| Pigg et al. [34] | Face: 0.09 ^o1^ | - | Face: 0.52 ^o5^ | - |
|  | Tongue: 0.63 ^o2^ | - | Tongue: 0.75 ^o6^ | - |
|  | Right Gingiva: 0.37 ^o3^ | - | Right Gingiva: 0.81 ^o7^ | - |
|  | Left Gingiva: -0.03 ^o4^ | - | Left Gingiva: 0.13 ^o8^ | - |
|  |  |  |  |  |
| **Clinical** |  |  |  |  |
| Baad-Hansen et al. [2] | GP: 0.04 ^p1^ | - | GP: 0.66 ^p4^ | CV: 38.3±24.4% |
|  | GNP: 0.63 ^p2^ |  | GNP: 0.13 ^p5^ |  |
|  | Hand: 0.31 ^p3^ |  | Hand: 0.57 ^p6^ |  |
| Brady et al. [4] | RA, leg – | - |  |  |
|  | WUD: 0.77 (0.39, 0.92) ^q1^ | LoA: -0.74 (-3.02, 1.53) |  |  |
|  | WUR: 0.56 (0.13, 0.81) ^q2^ | LoA: 1.90 (14.83, 18.62) |  |  |
|  | LBP, forearm – |  |  |  |
|  | WUD: 0.78 (0.56-0.90) ^q3^ | LoA: 0.01 (-1.60, 1.61) |  |  |
|  | WUR: 0.71 (0.45-0.86) ^q4^ | LoA: 2.61 (-12.00, 17.22) |  |  |
| Dams et al. [8] | 0.62 (0.33, 0.80) ^r1^ | SEM: 1.108  LoA: -0.17 (-3.27, 2.93) | 0.58 (0.29, 0.78) ^r2^ | SEM: 1.291  LoA: -0.33 (-3.91, 3.24) |
| de la Coba et al. [9] | FM: 0.73 (0.46 – 0.86) ^s1^ |  |  |  |
|  | RA: 0.49 (0.07 – 0.76) ^s2^ |  |  |  |
| de Oliveira et al. [10] | - | - | Hand: 0.90 (0.78 – 0.96) ^t1^ | CV:71.05% |
|  |  |  | Back: 0.89 (0.76 – 0.95) ^t2^ | CV: 72.36% |
| Middlebrook et al. [28] | - | - | Local: 0.57 (-0.07 – 0.84) ^u1^ |  |
|  |  |  | Remote: 0.62 (-0.04 – 0.86) ^u2^ |  |
| Othman et al. [32] | Shoulder: 0.73 (0.57 – 0.84) ^v1^ | SEM: 0.50  LoA: -0.26 (1.84, 1.33) |  |  |
|  | Forearm: 0.65 (0.45 – 0.79) ^v2^ | SEM: 0.52  LoA: -0.19 (-1.77, 1.39) |  |  |
| Sachau et al. [37] | 0.7 CMS: -0.002 ^w1^ |  | 0.7 CMS: 0.24 ^w3^ |  |
|  | Neurotip: 0.09 ^w2^ |  | Neurotip: 0.70 ^w4^ |  |
|  |  |  |  |  |
| **Mixed** |  |  |  |  |
| Knox et al. [21] | 0.81 ^x1^ | - | Session A: 0.74 ^x2^ | - |
|  |  |  | Session B: 0.90 ^x3^ | - |
|  |  |  |  |  |

ICC: intraclass correlation coefficient; CV: coefficient of variation; CR: coefficient of repeatability; LoA: Bland-Altman limits of agreement; SEM: standard error of measurement; WUD: wind-up difference; WUR: wind-up ratio; GP: gingiva of painful tooth; GNP: gingiva of non-painful tooth; AT: anterior temporalis; TMJ: temporomandibular joint; TA: tibialis anterior; ULR: right upper limb right; ULL: left upper limb; LLR: right lower limb; LLL: left lower limb; SR: right of spine; SL: left of spine RA: rheumatoid arthritis; LBP: low back pain; FM: fibromyalgia; superscript (e.g., a1, b1,…x3) correspond to studies in forest plots.
