# Supplementary material for: Reliability of Temporal Summation of Pain in Healthy and Clinical Populations: A Systematic Review and Meta‐Analysis
Source: Eur J Pain. 2025 Aug 8;29(8):e70097. doi: 10.1002/ejp.70097 (PMC12333475; doi:10.1002/ejp.70097)
Supplement: Supplementary file 5 — Table S2: ejp70097‐sup‐0005‐TableS2.docx. [file EJP-29-0-s001.docx]

**Table S2. Reliability of single and repeated stimulus**

|  |  | **Single-Stimulus** | | | | **Repeated-Stimulus** | | | |
| --- | --- | --- | --- | --- | --- | --- | --- | --- | --- |
|  |  | **Within-session** | | **Between-session** | | **Within-session** | | **Between-session** | |
| **Study** | **Group** | **ICC** | **Response stability** | **ICC** | **Response stability** | **ICC** | **Response stability** | **ICC** | **Response stability** |
| Biurrin Manresa et al.[3] | NWR | - | - |  |  | - | - |  |  |
|  | Test 1 & 2: |  |  | 0.82 ^y1^ | CV: 16.8% |  |  | 0.80 ^y7^ | CV: 14.8% |
|  | Test 2 & 3: |  |  | 0.85 ^y2^ | CV: 14.4% |  |  | 0.84 ^y8^ | CV: 13.4% |
|  | Test 1 & 3: |  |  | 0.71 ^y3^ | CV: 22.0% |  |  | 0.62 ^y9^ | CV: 22.4% |
|  | EP | - | - |  |  |  |  |  |  |
|  | Test 1 & 2: |  |  | 0.91 ^y4^ | CV: 11.4% | - | - | 0.81 ^y10^ | CV: 12.7% |
|  | Test 2 & 3: |  |  | 0.94 ^y5^ | CV: 9.4% |  |  | 0.85 ^y11^ | CV:12.5% |
|  | Test 1 & 3: |  |  | 0.84 ^y6^ | CV: 15.2% |  |  | 0.68 ^y12^ | CV:18.8% |
| Dams et al. [8] |  | 0.69 (0.44, 0.84) ^z1^ | SEM: 1.157  LoA: -0.29 (-2.55, 3.68) | 0.55 (0.24, 0.76) ^z2^ | SEM: 1.31  LoA: -0.12 (-3.37, 3.86) | 0.88 (0.77, 0.94) ^z3^ | SEM: 0.882  LoA: -0.12 (-2.22, 2.68) | 0.86 (0.68, 0.92) ^z4^ | SEM: 0.963  LoA: -0.04 (-2.60, 2.74) |
| Sachau et al. [37] | 0.7 CMS | 0.75 ^aa1^ | - | 0.74 ^aa3^ | - | 0.91 ^aa5^ | - | 0.80 ^aa7^ | - |
|  | Neurotip | 0.86 ^aa2^ | - | 0.69 ^aa4^ | - | 0.91 ^aa6^ | - | 0.86 ^aa8^ | - |
| Vuileumier et al. [40] | Session 1 – |  |  | - | - |  |  | - | - |
|  | Test 1 & 2: | 0.98 (0.97, 0.99) ^bb1^ | CV: 6.4%  CR: 1.3 |  |  | 0.98 (0.98, 0.99) ^bb8^ | CV: 6.4%  CR: 1.0 |  |  |
|  | Test 2 & 3: | 0.99 (0.99, 0.99) ^bb2^ | CV: 4.7%  CR: 1.0 |  |  | 0.99 (0.99, 0.99) ^bb9^ | CV: 4.7%  CR: 0.7 |  |  |
|  | Test 1 & 3: | 0.97 (0.96, 0.98) ^bb3^ | CV: 7.8%  CR: 1.6 |  |  | 0.98 (0.96, 0.98) ^bb10^ | CV: 7.8%  CR: 1.2 |  |  |
|  | Session 2 – |  |  | - | - |  |  | - | - |
|  | Test 1 & 2: | 0.99 (0.97, 0.99) ^bb4^ | CV:5.6%  CR: 1.0 |  |  | 0.99 (0.98, 0.99) ^bb11^ | CV:5.6%  CR: 0.8 |  |  |
|  | Test 2 & 3: | 0.99 (0.98, 0.99) ^bb5^ | CV: 5.8%  CR: 1.1 |  |  | 0.99 (0.99, 1.00) ^bb12^ | CV: 4.7%  CR: 0.7 |  |  |
|  | Test 1 & 3: | 0.97 (0.94, 0.98) ^bb6^ | CV: 8.0%  CR: 1.4 |  |  | 0.98 (0.97, 0.99) ^bb13^ | CV: 7.9%  CR:1.2 |  |  |
|  |  | - | - | 0.75 (0.64 – 0.83) ^bb7^ | CV: 25.8%  CR: 4.0 | - | - | 0.75 (0.65 – 0.83) ^bb14^ | CV: 25.8%  CR: 4.0 |

NWR: nociceptive withdrawal reflex; EP: electrical pain; CV: coefficient of variation; CR: coefficient of repeatability; SEM, standard error of measurement; LoA: Bland-Altman limits of agreement
