# Supplementary material for: Reliability of Temporal Summation of Pain in Healthy and Clinical Populations: A Systematic Review and Meta‐Analysis
Source: Eur J Pain. 2025 Aug 8;29(8):e70097. doi: 10.1002/ejp.70097 (PMC12333475; doi:10.1002/ejp.70097)
Supplement: Supplementary file 6 — Data S1: ejp70097‐sup‐0006‐DataS1.docx. [file EJP-29-0-s005.docx]

**Supplementary A: Search Strategy**

**Search keywords:**

1. temporal summation
2. wind-up
3. dynamic sensory testing
4. quantitative sensory testing

Pubmed (n=19 026):

(((((((Temporal summation) OR (TSP)) OR (QST)) OR (Temporal summation of pain)) OR (wind-up)) OR (windup)) OR (dynamic sensory testing)) OR (Quantitative Sensory Testing)

Embase (n=4697):
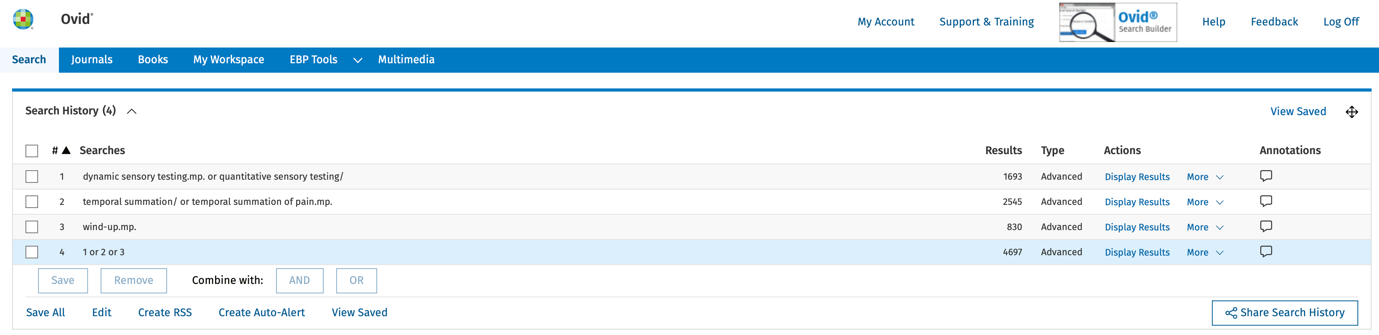


Web of Science (n=15624):

**
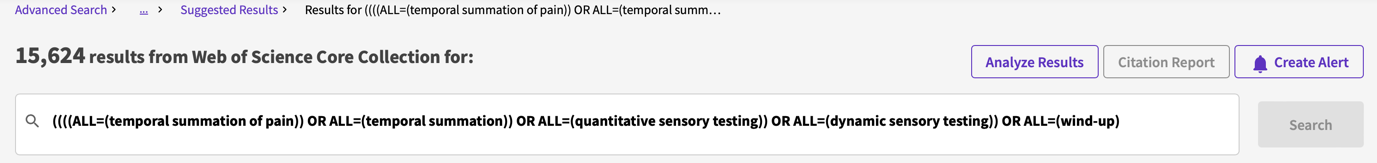
**
